# American Proctologic Society

**Published:** 1909-09

**Authors:** 


### Abstracts and Selections.

Reported for Texas Medical Journal.

### American Proctologic Society.

Eleventh annual meeting, held at Atlantic, N. J., June 7 and 8, 1909.

The President, Dr. Geo. B. Evans, of Dayton, Ohio, in the chair.

Officers elected for the ensuing year:

President—Dwight H. Murray, M. D., Syracuse, N. Y.

Vice President—T. Chittenden Hill, M. D., Boston, Mass.

Secretary-Treasurer—Lewis H. Adler, Jr., M. D., Philadelphia, Pa.

Executive Council—Geo. B. Evans, M. D., Dayton, Ohio, chairman; Dwight H. Murray, M. D., Syracuse, N. Y.; Louis J. Hirschman, M. D., Detroit, Mich.; Lewis H. Adler, Jr., M. D., Philadelphia, Pa.

The place of meeting for 1910 is St. Louis, Mo. Headquarters, Planters Hotel. June 6 and 7, 1910.

The following were elected fellows of the society: Dr. Chas. S. Gilman, 419 Boylston Street, Boston, Mass.; Dr. Donley C. Hawley, Burlington, Vt., and Dr. Frank C. Yeomans, 19 E. Forty-fifth Street, New York City, N. Y.

The following is an abstract of the President's address, "Progress in Proctology":

Who stated that not many years since, the creation of proctology as a specialty was frowned upon; for an indefinite period what was known of and what was done for diseases of the rectum was largely empiric, and not due to special knowledge or scientific study.

A few of us, at least, can remember when it was the rule among general practitioners to make no special effort to determine the pathology of diseases of the rectum; in fact, it was believed unbecoming the dignity of a high-classed, high-toned medical gentlemen to so lightly esteem modesty as to ask for the privilege of seeking the naked truth. Without attempting to make a diagnosis, opium and lead wash, with catharsis, was deemed sufficient treatment for any case. Little was taught in medical colleges of these diseases, for little was known and no special desire to learn much concerning them seemed to exist. But, fortunately, in the natural evolution of this specialty, this ignorance and indifference in the main, has been eliminated, and this field of work has assumed that of an accredited and justifiable specialty. No longer do we have to contend with the non-recognition of serious pathology, because of interposed modesty, ignorance and criminal indifference. A knowledge of the importance of being able to diagnose and treat intelligently diseases of the rectum is now considered essential for every general practitioner, and all this as a result of the creation of proctology by men who have made special effort to develop this field of work. The credit is due to such men as Adler, Allingham, Ball, Gripps, Edwards, Earle, Gant, Martin, Pennington, Kelsey, Matthews and others. To them are we indebted for progressive proctology.

As a matter of course, our pathology of this area is of necessity a modern pathology, and our knowledge of valves, varicosities, neoplasms, ulcerations and suppurations, are not based on hypothetical ideas of a quarter of a century since, but instead on the rather exact revelations of laboratory findings. The import of the presence of staphylococci, gonococci, colon bacilli and tubercle bacilli, is equally as much importance to the rectal surgeon as is the microscopical proof of the malignancy or benignity of a bit of tissue. With what greater assurance the proctologist approaches examinations of rectal diseases than did the physician of some years since. With a wide open field, if necessary, the aid of anesthesia, the proctoscope and the laboratory, there is usually not much difficulty in making a diagnosis,—a diagnosis inseparably linked with its dependents,—treatment and prognosis. Under the influence of progressive proctologic work ignorance and indifference to the recognition and treatment of rectal diseases is rapidly disappearing from the average medical man, as well as from the average layman. As a result of which the sum total of human suffering is immeasurably lessened, and individual existence is not so frequently abridged. The victims of rectal diseases are to be congratulated that this branch of science, or pseudo-science, has sufficiently advanced, that it now occupies the serious attention of the most progressive and intelligent men. The Lister methods of that day have been so changed and improved that they now seem very crude. The value of thorough cleanliness, asepsis, and the antiseptic influence of certain drugs, is of immeasureable value. It is now understood that the recto-anal area can be placed in a surgically clean condition, and that there need be no fear following operative interference. In not a few instances it obtains that relief is dependent on rectal surgery, when the subjects are unfit for narcosis produced from a general anesthetic, in cases of cardiac, pulmonic or nephritic diseases, making it hazardous to use general anesthesia. Sometimes it would seem that this danger of the uses of an anesthetic is too lightly thought of, and consequently, the mortality rate is increased. Local anesthesia, under cocaine infiltration, for the most part, is satisfactory, and is a great convenience to the operator and a life-saving narcosis in many instances.

The palliative treatment of hemorrhoids by proctologists is largely a matter of enforcement, viz.: where they are not permitted the opportunity to relieve by radical methods. The operative methods of removing hemorrhoids are so well understood, simple and effective, that it is foolish to attempt to relieve them by drugs or palliative measures.

The Allingham, or ligature method, when correctly and carefully performed, is generally applicable, but is not so free from pain and so quickly convalesced from as the clamp and cautery method. Many regard the last mentioned method as the one to be preferred. I believe, however, that the enucleation method approaches nearest to the ideal in results, and that the retention of the plug is not so painful as some would have us believe.

Proctoscopic examination is of importance, and is a distinct advance in rectal work. It is of great assistance in determining disease beyond discovery by ordinary methods. It is of distinct service in diagnosis, and of great value in aiding treatment in not a few conditions.

There is more hope for the ultimate cure of tubercular conditions; our better understanding of what environment means to these people will go far toward helping them to recovery, and there is not so much reason for a delayed recognition of the condition, which is of paramount importance.

I believe there is possibly a better understanding of syphilitic conditions, ulcerations, infiltrations and strictures, but the eternal dependence on anti-syphilitic treatment to resolve hyperplastic tissue is not so conspicuous, and progressive workers in this field realize that incision and excision are often necessary.

Concerning malignant and benign growths, the surgical rules that apply in other anatomical regions apply here. Early discovery and early removal is the only hope, as we all know, in malignant conditions, and as an advance, the removal of cancerous growths not within easy reach from below may be dealt with from above, or supra-pubicly, and just here it may not be inopportune to remark that it is to be believed that ere long it will be realized by the average physician that the removal of the rectum per se, is not as disastrous a matter as it is sometimes made to appear, especially since it is known that muscular transplantation will preserve more or less perfectly the function of the sphincters. The development of the technic essential to produce sphincteric power will relieve rectal extirpation of one of its most unpleasant features and render less hesitant many sufferers who should have the benefit of the operation.

Another matter of progressive interest is that colonic or rectal ptosis is amenable to intra-pelvic or intra-abdominal fixation, bringing relief that in some instances can not be hoped for by any other method of interference.

After all, the most encouraging sign is that the profession recognizes the fact that proctologists have a legitimate right to exist as specialists, and that diseases in the ano-rectal region deserve the same consideration as elsewhere. With the elimination of indifference, estheticism, modesty, the more universal belief in the necessity of early examination and diagnosis, we can but hope for greater progress and more relief to suffering humanity.

Gentlemen, when I consider the personnel of this Association, I am quite confident of the perpetuity of proctology as a distinct entity and am equally sure the progression in this special field of work will be in keeping with that in other specialties.

